# Clients' satisfaction of maternity care at a public referral teaching hospital, in Rwanda: case of University Teaching Hospital of Butare (CHUB)

**DOI:** 10.11604/pamj.2022.41.150.31421

**Published:** 2022-02-21

**Authors:** Emmanuel Habimana, Gaston Nyirigira, Augustin Sendegeya, Jules Ndoli Minega, Felicite Mukamana, Christian Ngarambe, Evariste Zigiranyirazo, Elias Rwamugema, Theogene Twagirumugabe

**Affiliations:** 1 Department of Obstetrics and Gynecology, University Teaching Hospital of Butare, Huye, Rwanda,; 2 Department of Anesthesia and Critical Care, University Teaching Hospital of Butare, Huye, Rwanda,; 3 The University Teaching Hospital of Butare, Huye, Rwanda

**Keywords:** Clients, satisfaction, maternity care, University Teaching Hospital of Butare, cross sectional study

## Abstract

**Introduction:**

maternal satisfaction is the key in health facilities utilization and so improving the birth outcome and reducing maternal morbidity and mortality. The main objective of this study was to assess women´s satisfaction with perinatal care provided in maternity at CHUB with its associated factors.

**Methods:**

a cross-sectional study was done at maternity of CHUB on clients´ satisfaction of maternity care. With a sample size of 422 mothers who were admitted for labor and delivery from July 1^st^ to October 31^st^ 2020. Data were collected using a structured questionnaire and client's satisfaction (eight questions (CSQ-8). Every respondent had to answer all questions under guidance of a data collector. Excel, Stata and SPSS were used for data entry and data analysis. Chi-squared and multivariate regressions were used for analysis of the association.

**Results:**

eighty-nine point thirty four (89.34%) percent of our respondents reported that services they received helped them to deal more effectively with their problems; and they were most satisfied with a mean score of 3.9 (97.5%) and least satisfied with the fact that they were not allowed to decide themselves in their management, with a mean score of 3.1 (77.5%). The overall satisfaction of our respondent’s equals to the mean score is 28.4/32= 88.75%. Factors found to affect mothers´ satisfaction were respecting mother´s privacy & values and allowing them to take decision and consenting before procedure.

**Conclusion:**

the majority of mothers were satisfied with received services. Respecting patients´ privacy and allowing them to participate in decision-making were two factors associated with high satisfaction.

## Introduction

Maternity care as healthcare related to pregnancy, childbirth and the post-partum period is very important in health facilities. Conducting patients´ satisfaction surveys in daily practice can give a general patients perception on services provided to them. These surveys help to unearth gaps in the quality of care delivered. Expectations and views from clients are of key importance for improved quality of service [1]. Maternal satisfaction may drive the maternal health facilities´ utilization, which can be linked, to the birth outcome and the reduction of maternal morbidity and mortality [2]. Maternal morbidity and mortality is still a big challenge in a safe motherhood [3]. Therefore, its reduction remains a priority worldwide as per the Millennium Development Goals 3 and 5 (promote healthier lives to all through investment in health systems) of sustainable development goals [4]. There is a need of a quality service delivery in general and in maternity services in particular to expect the decrease of maternal morbidity and mortality. With this achieved, clients´ satisfaction will increase as well as the fidelity to healthcare facilities as proven in a study conducted in Nairobi-Kenya in 2009 where 56% women would both like to recommend or deliver in the same facility basing on their previous satisfaction on the care received in the facility [5,6].

In Iran, change *et al*. in their study on satisfaction of maternity care in Lorestan Province found that the highest satisfaction score was related to confidentiality of the information, trusting the midwife, and cleanliness and hygiene of the delivery room while the lowest satisfaction was related to respecting silence in the pain room [7]. On the other hand, Melese *et al*. in their study done in 2014 on assessment of client satisfaction in labor and delivery services at a maternity referral hospital in Ethiopia revealed that the proportion of mothers who were completely satisfied with health care ranged between 2.4 to 21% and provider's communication with clients yielded complete satisfaction rates ranging between 0.7 to 26%. Inadequate information about the drug prescribed and explanation of procedures to be done to the client were found to be other major causes of dissatisfaction [8]. Levi D in 2005 in his study on the experience of women with vaginal examination and found that satisfaction index score was 74%, with factors such as privacy, dignity, sensitivity, support and frequency with which vaginal examinations were associated with women satisfaction [9]. Continuous and personalized care provided by the usual midwife and delivered within a family or a specialized setting, are also among factors influencing women´s satisfaction of maternity care [10]. In Rwanda, satisfaction on health services has been gradually increasing from 64.1% in 2014 to 77.4% in 2015 [11]. However, there is a paucity of published studies on maternity satisfaction in Rwanda. And our hypothesis was that clients seeking maternity care in the department of obstetrics and gynecology at University Teaching Hospital of Butare are not satisfied. The specific objectives of this study are to assess women´s satisfaction of maternity care provided to them and also to identify areas of high satisfaction and areas requiring improvement, and to determine factors influencing their satisfaction in maternity at a Public University Teaching Hospital of Butare, in Rwanda.

## Methods

This is a cross-sectional study carried out at a University Teaching Hospital of Butare (CHUB), one of public and referral hospitals in Rwanda, located in southern province of the country. CHUB is one the largest referral hospitals in Rwanda and it is receiving referrals from eighteen district hospitals, covering nearly a population of 3.5 million people. It has got different departments according to different specialties. The obstetrics and gynecology department is equipped with 86 beds and it has got a busy maternity as it is admitting around 3000 deliveries per year including 52% cesarean sections. It included all pregnant women who came to CHUB/maternity unit for labor or for scheduled cesarean section within the study period, literate and who accepted to voluntarily participate regardless the gestation age and regardless of their parity and living children. Systematic random sampling method was used to select subjects for our sample. Participants were recruited from the maternity admission every day the night as for the day. To reduce bias and to give an equal chance to all participants to be selected; for every two clients of those who gave their consent to participate in this study an explained questionnaire was given by a data collector at the admission and this questionnaire was submitted at discharge day. Our sample size was calculated to be 384 using the following formula:


$$n\geq Z^{2}(p)(1-p)/d^{2}$$

Where, n: the minimum size required; Z: Z- score corresponding to the level of confidence with which it is desired to be sure that true population lies with ± d% point of the sample estimate (Z=1.96); P: expected population proportion (P=0.50); e: margin of error (0.05). We added 10% randomly to cover the loss of questionnaires during the hospital stay and the exact sample size became 422 clients.

Data was collected using a structured questionnaire and the subjects had to answer all questions under guidance of a research assistant. Research assistants had a training of one day before starting data collection. They were assigned in different obstetrical wards and at admission to handle any concern on the questionnaire from respondents. To test the questionnaire reliability and validity, its content was reviewed and approved by experts in research. A sample of thirty copies was given to patients and health care providers for testing if any concern on the structure and content of the questionnaire. Questions that caused confusion in answering were rephrased or removed. The CSQ-8 instrument, with some modifications made on it, was used for assessing client satisfaction. The CSQ-8 is a brief, standardized measure of client satisfaction that is comprised of eight items and all items are scored using a four-point scale. The total score ranges from 8-32 scores, and the higher score the more the client is satisfied [12]. Data was collected from July 1^st^, 2020 to October 31^st^, 2020 by four data collectors. Research assistants were healthcare providers hired and trained on the questionnaire for the purpose of data collection in this study. Database was created using Microsoft Excel and was exported into STATA 13 for analysis. Chi-squared tests and logistic regression were used for analysis for comparison and correlation between clients´ satisfaction, factors associated with satisfaction and dissatisfaction. P<0.05 was considered for significant statistical difference. Before carrying out this study, the ethics approval number RC/UTHB/035/2018 was obtained from the CHUB institution review board (IRB). For every single client enrolled in the study, her written and signed consent was obtained before completing the data collection form. To ensure confidentiality of data given by a client, data collection forms was not containing any identifier and while collecting data each data collection form was filled in by a client herself guided by a data collector.

## Results

The sample size of this study was made up of four hundred and twenty-two clients who came in for labor and delivery and all were meeting the inclusion criteria. There were no missing data as research assistants were always there to collect all filled questionnaire, so all questionnaires were analysed. Majority of respondents were in 25-35 years age group (68.72%) and were married (97.6%). For their education level, most of them attended university and higher education (41.67%) and those who attended secondary school were 36.7%. They were mostly public servants (32.5%) followed by private business women (31.6%). About their health insurances, majority of our respondents were having Rwanda Social Security Board (RSSB) (45.73%) followed by Community Based Health Insurance (CBHI) (39.34%). And they were mostly between 1-2 parity (51.2%) (Table 1).

**Table 1:** sociodemographic characteristics of respondents

Variable	Value	Count	Percentage
Age	<20	4	0.95
	20-25	44	10.43
	25-30	146	34.60
	30-35	144	34.12
	>35	84	19.91
Level of education	None	7	1.66
	Primary	87	20.62
	Secondary	153	36.26
	University and higher education	175	41.47
Marital status	Single	4	0.95
	Married	412	97.63
	Divorced	5	1.18
	Widow	1	0.24
Occupation	Private	129	30.57
	Public	141	33.41
	Student	20	4.74
	Farmer	88	20.85
	Other	44	10.43
Health insurance	None	5	1.18
	CBHI	166	39.34
	RSSB	193	45.73
	BRITAM	10	2.37
	MMI	24	5.69
	MIDPLAN	16	3.79
	RADIANT	0	0
	UAP	1	0.24
	Other	7	1.66
Parity	0	21	4.99
	1-2	215	51.07
	3-4	124	29.45
	4--5	39	9.26
	>6	22	5.21

About the overall satisfaction on received services, meeting their expectations and future services utilisation, this study showed that 377/422 (89.34%) of respondents received services that helped them to deal more effectively with their problems, 265/422 (62.80%) reported that they were very satisfied with received services while 118/422 (27.96%) were mostly satisfied. 298/422 (70.62%) reported to definitely come back for the same service, 114/422 (27.01%) think they can come back to the service and 329/422 (78.33%) would like to definitely recommend the service to their friends while 79/422 (16.67%) think they may recommend it to their friends (Table 2). About the appreciation of maternity wards environment in the current study, 54.61% of respondents appreciated the silence in labor and delivery room, 74.23% appreciated the light, ventilation and warmness in delivery room, 73.29% appreciated the comfort with bed in delivery room and waiting room and 76.36% appreciated the cleanliness in delivery and post-partum rooms. Through the CSQ-8 tool to further assess client’s satisfaction, findings of this study show that respondents were least satisfied with the fact that they were not allowed to decide themselves in their management, with a mean score of 3.1 and this means they were satisfied with this at 77.5%. The patients were most satisfied with the fact that the services they received helped them to deal with their concern with a mean score of 3.9 (97.5%). And the overall satisfaction of our respondents equals to the mean score is 28.4/32= 88.75% (Table 3).

**Table 2:** clients’ satisfaction on received maternity services

Variable	Value	Count	Percentage
Have the services you received helped you to deal more effectively with your problems?	Yes, they helped a great deal	377	89.34
	Yes, they helped somewhat	44	10.43
	No, they seemed to make things worse	0	0
	No, they seemed to make things worse	1	0.24
In an overall, general sense, how satisfied are you with the service you received?	Very satisfied	265	62.80
	Mostly satisfied	118	27.96
	Indifferent or mildly dissatisfied	36	8.53
	Quite dissatisfied	3	0.71
If you were to seek help again, would you come back to the service?	No, definitely not	2	0.47
	No, I don't think so	8	1.90
	Yes, I think so	114	27.01
	Yes, definitely	298	70.62
Would you recommend the same service to a friend?	No, definitely not	5	1.19
	No, I don't think so	16	3.81
	Yes, I think so	70	16.67
	Yes, definitely	329	78.33

**Table 3:** client satisfaction on maternity care analysis using CSQ-8

Item	Mean(standard deviation)
Did received services help you deal with your concern or not?	3.9(0.3)
At which level were you satisfied from received services?	3.5(0.7)
Will you return for the same services?	3.7(0.5)
Would you recommend the service to your friends?	3.7(0.6)
Did medical doctors take your concern seriously?	3.5(0.7)
Did you get a help when needed a health care provider?	3.4(0.7)
Did medical doctors respected your views and decision?	3.6(0.6)
Did you decide yourself for your management?	3.1(1)
Total (mean)	28.4

After multivariate logistic regression, factors found to affect mothers´ satisfaction were respecting mother´s privacy and values and allowing them to take decision and consenting before procedure. Mothers with respected privacy and values were 10 times more likely to be satisfied than mothers whom their privacy and values were not respected (AOR: 10.3, 95%CI: 1.2-86.7). Mother involved in decision-making and consented before procedure were 8 times more likely to be satisfied than those who were not involved (AOR: 8.5, 95% CI: 1.8-41.2) (Table 4). When asked about what they would like to be changed by CHUB maternity unit for better quality of care. Maternity ward hygiene in general, waiting time with patient flow, the way some midwives and medical staff communicated with them, midwives and receptionist behaviours and lack of essential drugs in the hospital´s pharmacy were among other points with which some respondents were not satisfied with 28.44% of respondents wished an improvement in hygiene in rooms, shortening the payment process was reported by 16.11% of our respondents, increase of water supply in maternity unit was reported 6.87%, improvement of communication towards caretakers and patients was reported by 6.16%, improve of availability of essential drugs as reported by 6.16% of our respondents and lastly but not least, increase of doctors in weekends as suggested by 7.1%.

**Table 4:** factors influencing mother’s satisfaction (multivariate logistic regression)

Variable	Category	AOR	95%CI	p-value
Light, ventilation and warmness in delivery room	Yes/No	3.5 reference	0.7-17.3	0.126
Helpfulness of received services	Yes/No	1 reference		
Satisfied from received services	Yes/No	0.5 reference	0.1-3.6	0.469
Returning for the same services	Yes/No	7.4 reference	0.2-263.6	0.271
Recommending the service to others	Yes/No	0.2 reference	0.01-6.0	0.357
Respected mother's dignity from reception	Agree/Disagree	2.2 reference	0.3-15.8	0.441
Taking mother's concern seriously by MD	Agree/Disagree	0.9 reference	0.04-24.3	0.977
Presence of needed healthcare providers	Agree/Disagree	4.3 reference	0.2-85.3	0.335
Respecting mother's value and privacy	Agree/Disagree	10.3 reference	**1.2-86.7**	0.031*
Abused mother during service delivery	Agree/Disagree	1.2 reference	0.15-9.5	0.857
Allowing mother to take decision and consenting before procedure	Agree/Disagree	8.5 reference	**1.8-41.2**	0.008*

Variables found to be statistically significant were filtered for multivariate logistic regression and corresponding adjusted odd ratio with their respective 95% confidence intervals were determined

## Discussion

This is the first study on clients´ satisfaction of maternity care in a public referral teaching hospital in Rwanda. The main purpose of this study is to assess women´s satisfaction of maternity care provided to them and also to identify areas of high satisfaction and areas requiring improvement, and to determine factors influencing their satisfaction in maternity at a Public University Teaching Hospital of Butare, in Rwanda. The overall satisfaction of received services using CSQ-8 tool were also analyzed. In this study, the demographic characteristics of our respondents were similar compared to other studies on the same topic with a little difference on health insurances depending on the country and the place where the study was done [13,14]. More than a half of respondents, 68.72% (n=422) were of young age (25-35 years old) and few of them were not married (2.4% of respondents). The majority of our respondents were of at least secondary level of education, and this is explained by the fact that participants in this study had to be literate. Majority of our respondents were having health insurances (mainly public health insurances: Rwanda Social Security Board (RSSB) and Community Based Health Insurance (CBHI)) and this is allowing them to use health services as health services are mostly being used when users are having health insurance that can help them to cover the cost of provided health care services [14,15]. In the same line of the health facility utilization, studies showed that mothers use maternity services when they are satisfied and mainly when they are in specialized and affordable setting with specialist obstetricians, with availability of doctors, or with usual staff or when services are available in their proximity. And from results of this study, mothers are coming to maternity of CHUB because they like the hospital staff with the presence and availability of obstetricians and gynecologists specialists or because CHUB is the only convenient hospital to them. Studies have also shown that clients´ satisfaction of maternity care and health facility utilization are linked with the maternity environment, clients´ perception and appreciation on maternity wards. The current study showed the similar findings with three studies [16-18] as most of the respondents appreciated positively the silence, the light, ventilation and warmness, the comfort with bed and the cleanliness in labor, delivery and postpartum rooms.

This study highlights the fact that clients use or seek maternity services when they expect those services will deal more effectively with their problems and when received services meet their expectations they may come back, or recommend them to their friends or family member. In this study, for 89.34% of respondents, the services they received helped them to deal more effectively with their problems; 62.80% were very satisfied with the services they received while 27.96% were mostly satisfied. 70.62% reported to definitely come back for the same service, and 78.33% would like to definitely recommend the service to their friends. And the overall satisfaction of our respondents equals to the mean score is 28.4/32 (88.75%). Our results are similar to those from studies done in 2009 in Kenya, in Iran in 2015, in Nepal in 2018 and in 2019 in Mozambique where most of the respondents were respectively satisfied with maternity care and would like to recommend their friends or family members to sick the maternity services in the same hospital [2,5,6,18-20]. Attitude and communication of health care providers in labor and delivery rooms, and of clerks and receptionists, are independent predictors of mothers´ satisfaction. In current study, clerks and receptionists treated clients with dignity, medical doctors took their questions and concerns seriously, health care providers were present especially when needed most, and medical doctors respected their values and privacy, and most of the clients witnessed absent physical abuse/harassment/cursing words, anger and poor conducts from health care providers.

However, most health care providers in maternity ward did not introduce themselves in their names, qualification and expertise and some medical doctors did not allow clients to make decisions and get their consent before any procedure or exam. This has a negative impact on clients´ satisfaction and contribute to their dissatisfaction as shown also by different studies done in Bangladesh, two studies in Ethiopia, in Rwanda, and Nigeria [1,8,14,16,19]. The current study showed that the presence of comfortable labor and delivery rooms, meeting clients expectations, respecting mother´s dignity, values and privacy, allowing mother to participate in decision-making and consenting before any procedure were factors influenced the clients' satisfaction. Mothers with respected privacy and values were 10 times more likely to be satisfied than mothers whom their privacy and values were not respected. Mother involved in decision-making and consented before procedure were 8 times more likely to be satisfied than those who were not involved. Findings of this study are similar to findings of other various studies that highlighted the role of receptionists, understanding mothers concerns seriously, availability of health care providers when needed most, confidentiality, respect and involving the mothers in the decision in their management as among other predictors of clients´ satisfaction [20-22]. This study assessed areas that require improvement for high quality of maternity and its findings showed that there is a need of improving maternity ward hygiene in general, decreasing waiting time with patient flow, changes on the way some midwives and medical staff communicate with patients and caretakers, improving midwives and receptionist behaviors and availing essential drugs in hospital´s pharmacy. Those findings are similar to results of other studies like the one done in Nepal in 2015 in which hygiene/cleanness was a major concern to both side satisfied and not satisfied clients and various studies highlighted the role of current maternal experience to the future use of maternal services [16,17,20,22].

**Study limitations:** this study did not assess the association between the delivery outcome, labor pain management and clients’ satisfaction. Illiterate clients did not have a chance of participating in this study, and this may lead to missing important information on their satisfaction. Data of this study cannot be generalized to appreciate the clients’ satisfaction of health care delivered to them at the level of hospital, rather it may give a general picture as pilot study.

## Conclusion

Majority of mothers were satisfied of received services, however in overall satisfaction some mothers neither were satisfied nor dissatisfied. This study showed that respecting clients’ values and privacy and involving them in decision-making are key factors for satisfaction of maternity care at CHUB. We recommend carrying out a study in overall hospital to see how much clients are satisfied with the services they are receiving in all health care departments.

### What is known about this topic

- *Clients satisfaction is associated with health facility use;*
- *Attitude and communication of health care providers towards clients are independent predictors of clients’ satisfaction;*
- *Pain management and usual health facility or usual health care providers are associated with higher satisfaction*.

### What this study adds

- *The presence of comfortable labour and delivery rooms, respecting mothers' dignity, values and privacy were associated with high clients' satisfaction*.
